# Nanotechnology for the theranostic opportunity of breast cancer lung metastasis: recent advancements and future challenges

**DOI:** 10.3389/fbioe.2024.1410017

**Published:** 2024-05-31

**Authors:** Lin Miao, Yue Kang, Xin Feng Zhang

**Affiliations:** Departemnt of Breast Surgery, Cancer Hospital of China Medical University, Cancer Hospital of Dalian University of Technology, Liaoning Cancer Hospital and Institute, Shenyang, China

**Keywords:** breast cancer, lung metastasis, nanotechnology, theranostic, drug delivery

## Abstract

Lung metastasis of breast cancer is rapidly becoming a thorny problem in the treatment of patients with breast cancer and an obstacle to long-term survival. The main challenges of treatment are the absence of therapeutic targets and drug resistance, which promotes the development of nanotechnology in the diagnosis and treatment process. Taking advantage of the controllability and targeting of nanotechnology, drug-targeted delivery, controlled sustained release, multi-drug combination, improved drug efficacy, and reduced side effects can be realized in the process of the diagnosis and treatment of metastatic breast cancer (MBC). Several nanotechnology-based theranostic strategies have been investigated in breast cancer lung metastases (BCLM): targeted drug delivery, imaging analysis, immunotherapy, gene therapy, and multi-modality combined therapy, and some clinical applications are in the research phase. In this review, we present current nanotechnology-based diagnosis and treatment approaches for patients of incurable breast cancer with lung metastases, and we hope to be able to summarize more effective and promising nano-drug diagnosis and treatment systems that aim to improve the survival of patients with advanced MBC. We describe nanoplatform-based experimental studies and clinical trials targeting the tumor and the tumor microenvironment (TME) for BCLM to obtain more targeted treatment and in the future treatment steps for patients to provide a pioneering strategy.

## 1 Introduction

With the increasing incidence, breast cancer has become the most common malignant tumor in females. For women, breast cancer will account for one-third of new malignancies in 2022 and is one of the leading causes of death worldwide (Siegel et al., 2022). The prognosis of breast cancer has been greatly improved with more and more treatment options available, but metastasis is still the cause of poor prognosis and death. According to related reports, about 20%–30% of breast cancer patients may metastasize after the corresponding diagnosis and treatment (Cancer Genome Atlas, 2012). Breast cancer metastases usually occur in the lungs, liver, bone, and brain. The lung is the second most metastatic site of breast cancer, and more than 60% of breast cancer patients develop lung metastases in the advanced stage. Once the tumor has metastasized to distant organs, MBC is largely untreatable due to limited treatment options, and survival after 5 years is only about 20%, however, the overall 5-year survival rate of patients with BCLM is even only 16.8%. The data demonstrates that 60%–70% of patients who die from BCLM (Schlappack et al., 1988; Smid et al., 2008; Nguyen et al., 2009; Eckhardt et al., 2012; Lee et al., 2019; Kim et al., 2021). Despite ongoing efforts by researchers to treat breast cancer, overcoming advanced tumor metastasis is still a great challenge in cancer therapy, which often leads to treatment failure. Therefore, it is a difficult but clinically significant direction in the field of tumor therapy to explore the related treatment for patients of incurable BCLM.

Tumor metastasis is a process involving multiple organs and various cell biology, including the transfer of tumor cells from one organ to another and adaptation to a new TME. Each step of tumor metastasis needs multi-genes and multi-factors to participate in a comprehensive role. At the same time, tumor metastasis is closely related to tumor proliferation, apoptosis, invasion, migration, angiogenesis, and other related genes as well as related signaling pathways (Carmeliet and Jain, 2011). The metastasis of breast cancer to the lung is a multi-layered and complex process, which is influenced by many factors. Relevant data suggest that the incidence of lung metastases in breast cancer varies by molecular subtype, among which triple-negative breast cancer (TNBC) is highly aggressive, approximately 46% of patients with TNBC develop distant metastases, and lung metastases occur in 21%–32%, whereas the median overall survival after metastasis in BCLM is only 13.3 months (Bianchini et al., 2016; Schmid et al., 2018; Medeiros and Allan, 2019; Yin et al., 2020). To clarify the mechanism of lung metastasis of breast cancer and to provide effective targets for precise therapy are the current research ideas of breast cancer metastasis.

At present, in the clinical treatment of breast cancer, the commonly used treatment means are surgery, endocrine therapy, chemotherapy and radiotherapy, and targeted therapy, immunotherapy, and other treatment methods are also being studied (Caswell-Jin et al., 2018; Dahan et al., 2023; Verma et al., 2024). Traditional treatment has a good effect on early breast cancer, but it has little effect on BCLM (Gennari et al., 2021).In addition to poor prognosis, these patients often have a variety of clinical symptoms, including cough, hemoptysis, pleural effusion, and pulmonary dysfunction, which profoundly affect patients’ quality of life and survival (Jin et al., 2018). The application of nanotechnology in the treatment of cancer has provided a new way of thinking about the treatment of cancer, which has gradually become the research hotspot of cancer treatment and made a lot of progress. Treatment of MBC using nanoparticle drug delivery systems has the potential to improve drug delivery, controlled release, and biocompatibility, thus improving drug efficacy and reducing side effects. It is possible to target multiple metastatic sites at the same time and to achieve better therapeutic effects and lower injury effects by combining multiple therapeutic methods (Silberholz et al., 2019; Sudheesh et al., 2022; Rahman et al., 2023). In this review, we attempt to outline the latest advances in the application of nanoscale diagnostic and therapeutic systems for BCLM, with particular emphasis on the mechanisms and research exploration. In addition to presenting experimental validation of BCLM, we discuss potential therapeutic approaches based on nanotechnology that may improve the prognosis of patients with BCLM.

## 2 Current treatment strategies and challenges of BCLM

Tumor metastasis generally consists of the following steps (Thiery, 2002): metastatic tumor cells shed from the primary tumor and infiltrate into the surrounding extracellular matrix (ECM), enter the blood or lymphatic vessels, invade and attack distal tissues or organs, settlement of new metastasis, and eventually form distal metastasis. In the process of lung metastasis of breast cancer, we can also regard metastatic breast tumor cells as “seeds,” and the microenvironment provides “soil” during the process of metastasis. “Seeds” are often involved in molecular and functional alterations, while “seeds”are also capable of substance secretion to enhance invasiveness and alter the tumor microenvironment to promote the formation of BCLM. For example, a study has explored that tumor-secreted glucocalcin 1(STC1) can upregulate the expression of S100 calcium-binding protein a 4(S100A4) by promoting the phosphorylation of EGFR and ERK signaling pathways in breast cancer cells to enhance the invasiveness of tumor cells and promote angiogenesis and activation of lung fibroblasts in the metastatic microenvironment, and ultimately promote lung metastasis of breast cancer (Liu et al., 2023). Cancer cell-derived secretory factors can secrete components such as hyaluronan, collagens, laminin and fibronectin via the ER-Golgi pathway and the Golgi-independent pathway, which induce ECM remodeling in the tumor microenvironment, promote cancer cell proliferation, and penetrate tissues and blood vessels, then it spreads to the lungs. Multiple molecules and cellular components secreted by both primary breast tumors and stromal cells, such as PD-L1, P2Y2R, PGE2, transforming growth factor-β (TGF-β), matrix metalloproteinases (MMPs), etc., mediate the complex network of tumor microenvironment, lung stroma, immune cells and bone marrow-derived cells (BMDCs) and provide a fertile niche susceptible for the promotion of breast cancer metastasis to the lung (Paltridge et al., 2013; Medeiros and Allan, 2019; Urooj et al., 2020). For example, Wang et al. (2023) found that Cav-I in BC-derived exosomes regulates the induction of ECM deposition by lung fibroblasts by upregulating PMN(pre-metastatic niche)-associated genes in lung epithelial cells; Inhibition of the alveolar macrophage PTEN/CCL2/VEGF-A signaling pathway to influence M2-type polarization and angiogenesis promotes the formation of a PMN and ultimately leads to breast cancer lung metastasis. In-depth study of the mechanisms of lung metastasis in breast cancer is essential to identify new biomarkers and explore potential therapeutic targets to provide new ideas for possible therapeutic modalities of BCLM. From the point of view of treatment, eradication of the primary tumor, blocking the spread and targeting the metastasis are the three main methods to reduce the tumor metastasis.

For early and middle stage solid tumors, surgical resection can greatly prolong the life span of patients and improve the survival rate of patients, but some studies have shown that surgery and surgical trauma can also promote the formation of new metastases and accelerate the development of micro-metastases (Tohme et al., 2017). Some researchers have reported that circulating tumor cells (CTCs) are associated with tumor metastasis and recurrence after surgery. Surgery inevitably damages the vasculature, disrupts the balance between the immune system and CTCs, and triggers a cascade of local and systemic inflammation. The primary tumor suppresses small metastatic lesions, leaving them in a “dormant state” that maintains a balance between proliferation and apoptosis, but this “homeostasis” is disrupted after surgery, at the same time, the decrease of angiogenesis inhibitor level in the traumatic blood vessels after operation leads to the rapid growth of metastatic lesions (Michelson and Leith, 1994; Valastyan and Weinberg, 2011; Chiarella et al., 2012; Horowitz et al., 2015; Xu et al., 2015). Although some retrospective studies have found that surgery may be a factor in prolonging the survival of MBC, case selection bias may be one of the major reasons for concluding patient benefit (Blanchard et al., 2008; Neuman et al., 2010; Poggio et al., 2018). More and more works of literature show the effect of surgical treatment on metastatic breast cancer, but the survival benefit of surgery has not been clearly established and the choice of surgery in MBC remains controversial (Thomas et al., 2016; Khan et al., 2022; Ren et al., 2024). Some relevant clinical studies (Badwe et al., 2015; Fitzal et al., 2019; Soran et al., 2021; Khan et al., 2022) on the impact of surgery on survival in patients with MBC are listed in Table 1. As far as the current criteria are concerned, it remains to be further discussed whether surgery should be performed for advanced MBC. For advanced metastatic breast cancer, the benefits of local radiotherapy are not well understood, while the lungs are poorly tolerant to radiation and radiotherapy is often palliative (Feys et al., 2015; Zhang et al., 2023). Recent studies have explored new treatment methods such as radiotherapy with hyperthermia, but only play a role in local tumor remission and symptom control.

**TABLE 1:** The relevant clinical studies on the impact of local and systemic treatment on survival in patients with metastatic breast cance are listed in Table 1.

Group	NCT/Study name	Group/Cohort	Sample size	Outcome
Local treatment	NCT00557986	locoregional treatment group and systemic therapy group	278	Breast locoregional surgery improves the local progression free survival
	NCT01242800	Early local therapy&Continued systemic therapy	256	Early locoregional therapy associated with improved local control, but this had no overall impact on quality of life
	NCT01015625	surgery and systemic Therapyandsystemic therapy without surgery)	90	Primary surgery could not demonstrate an OS benefit for breast cancer patients with *de novo* stage IV disease
	NCT00193778	locoregional treatment and no locoregional treatment	350	The trial failed to show an OS benefit from locoregional therapy
Systemic treatment	NCT03366844	single-arm	8	The combination of palliative RT with pembrolizumab did not demonstrate clinical activity in this group of patients with heavily pre-treated HR + MBC.
	NCT02730130	single-arm	17	The combination of pembrolizumab and RT was found to be safe and demonstrated encouraging activity in patients with poor-prognosis, metastatic, triplenegative breast cancer who were unselected for programmed death-ligand 1 expression
	IMpassion130	atezolizumab and nab-paclitaxel vs. placebo and nP in unresectable	451	Atezolizumab and nab-paclitaxel clinical benefit was only observed in patients whose tumors were PD-L1 IC+
	KEYNOTE-355	pembrolizumab plus chemotherapy vs. placebo plus chemotherapy	847	Among patients with advanced triple-negative breast cancer whose tumors expressed PD-L1 with a CPS of 10 or more, the addition of pembrolizumab to chemotherapy resulted in significantly longer overall survival than chemotherapy alone
	MONALEESA-2	ribociclib plus letrozole or placebo plus letrozole	668	The improved efficacy outcomes and manageable tolerability observed with first-line ribociclib plus letrozole are maintained with longer follow-up, relative to letrozole monotherapy
	PALOMA-2	palbociclib plus letrozole vs. placebo plus letrozole	666	A trend towards prolonged OS was seen favoring the combination and improvement in progression-free survival for palbociclib plus letrozole *versus* placebo plus letrozole in ER+/HER2– advanced breast cancer
	TROPION-Breast02	antibody–drug conjugate datopotamab deruxtecan vs. ICC	600	onngoing

Few patients with metastatic tumors can be cured by surgical intervention (Steeg, 2006), while systemic therapy has still been a routine treatment for MBC. The conventional methods of systemic therapy for MBC include chemotherapy, endocrine therapy, and molecular targeted therapy. Recent advances in systemic therapy have increased the survival rate of patients with MBC (Caswell-Jin et al., 2018), and traditional chemotherapy is one of the main methods of anti-tumor therapy at present. However, these anticancer drugs have blind-killing effects, can cause severe side effects and varying degrees of drug resistance, and are less effective against breast cancer metastasis and invasion. Some studies have shown that the median progression-free survival time of patients with locally advanced or advanced TNBC treated with first-line chemotherapy is only 5.6 months, and the rate of grade 3 or above related adverse events associated with chemotherapy is high (Cortes et al., 2020). Chemotherapy resistance is often the main cause of chemotherapy failure, especially in MBC and TNBC, and it even accounts for approximately 90% of treatment failures (Nedeljkovic and Damjanovic, 2019). More and more studies have revealed that the development of resistance to MBC chemotherapy is complex and is based on several factors, including the interaction of TME, drug efflux, cancer stem cells, and bulk tumor cells, while changes in multiple signaling pathways control these interactions (Mao et al., 2013; Boelens et al., 2014; Wang et al., 2018; Luo et al., 2020; Gao et al., 2021; Subhan, 2022). At present, in-depth study of chemotherapy drugs has also found their adverse mechanism of action. For example, although paclitaxel is clearly beneficial in reducing tumor size, it alters the lung microenvironment and promotes cancer cells colonizing the lungs (Chang et al., 2017; Keklikoglou et al., 2019). The era of molecular targeted rescue therapy for MBC has come. More and more clinical studies (Hortobagyi et al., 2018; Rugo et al., 2018; Emens et al., 2021; Cortes et al., 2022; Dent et al., 2023; Slamon et al., 2024)confirm the status of targeted drugs and the benefits of the combination of targeted drugs (Table 1). HER2-targeted therapy including mAb, ADC, and small molecule TKIs has important significance in HER2-positive metastatic breast cancer (Swain et al., 2020). PIK3CA mutations are common in metastatic ER-positive breast cancer and can be treated with PI3K inhibitors such as alpelisib (Mayer et al., 2017). PARP inhibitors (PARPI) have antitumor activity in advanced breast cancer and BRCA-mutant patients (Desnoyers et al., 2022). PD-1/PD-L1 inhibitors alone or in combination have been shown to be effective in the treatment of metastatic TNBC (Ren et al., 2022). The continuous updating of therapeutic drugs may change the treatment pattern and survival rate of MBC in the near future. In fact, most drug delivery systems can not precisely target the metastatic site, resulting in poor anti-metastatic effect, so it is important to find new effective metastasis targeting strategies to improve the therapeutic effect of cancer metastasis.

## 3 Role of nanoparticles in current cancer diagnosis and therapy

The existing methods of anti-tumor therapy have definite limitations and limited therapeutic effects. Therefore, it is necessary to find a new and effective way of drug delivery to improve the therapeutic effect of tumor metastasis. The nano-drug delivery system is a promising way to break through the dilemma (Schroeder et al., 2011). Compared with traditional drug dosage forms and therapeutic methods, drug delivery systems developed by nanotechnology and new therapeutic methods have unique advantages: increase the solubility of insoluble drugs and decrease their clearance rate; modification on the carrier platform can improve the drug circulation time in tumor and TME, and control the drug release process; the carrier system was modified to respond to the TME and achieve active drug targeting; combined therapy with multi-drug delivery can reduce the single drug resistance of tumor cells and improve the therapeutic efficiency; the drug delivery system was constructed to include imaging drugs for imaging and dynamic observation of tumor (Arvizo et al., 2011; Gurunathan et al., 2018; Palazzolo et al., 2018; Aghebati-Maleki et al., 2020).

**FIGURE 1:**
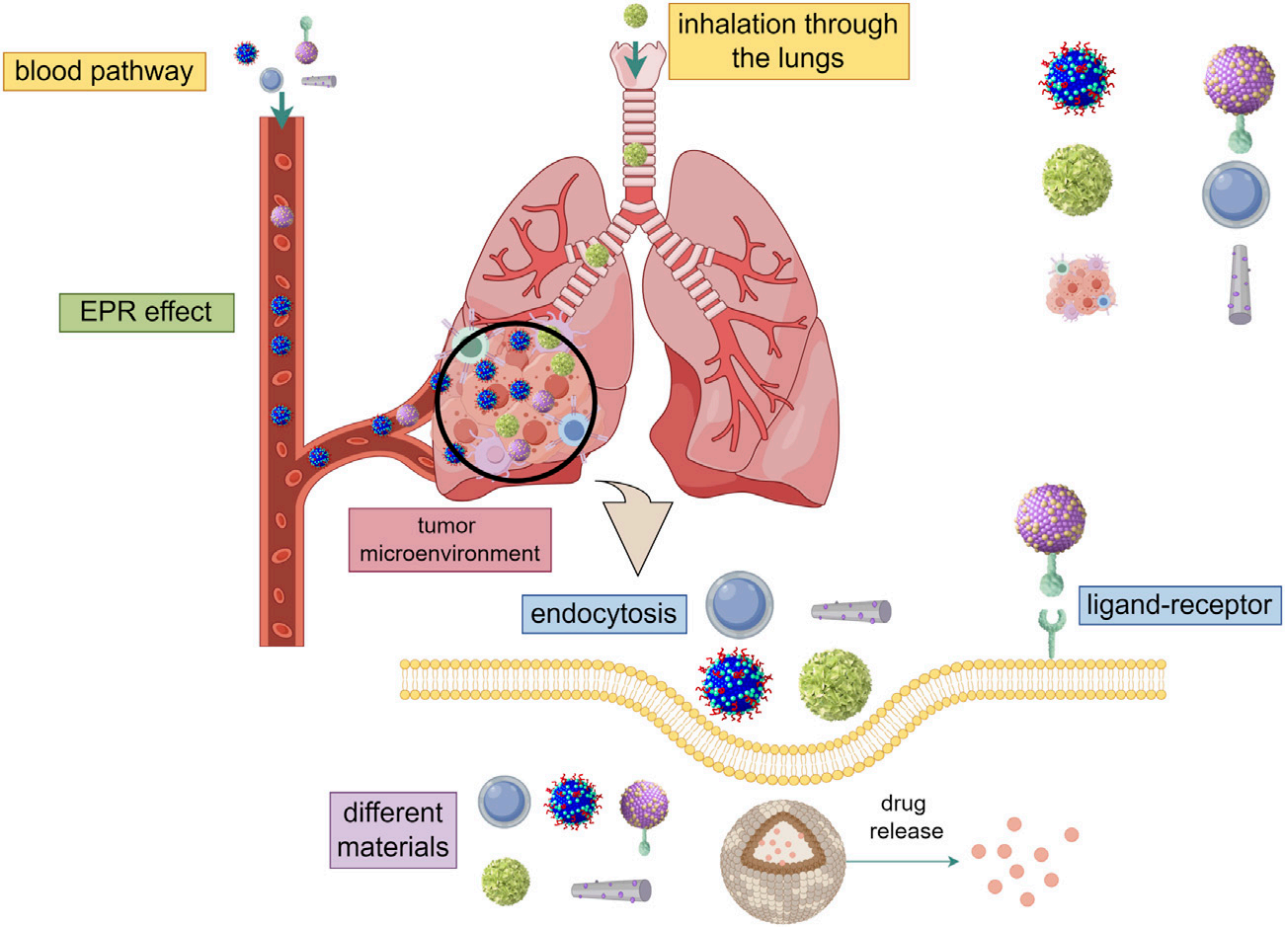
The transfer process and influencing factors (By Figdraw). ① Through intravenous injection and inhalation of two ways into the lung metastatic tumor, using EPR effect and charge reversal ability to avoid the elimination of drugs in the blood circulation when they are outside the cell. ② Entry into the cell by endocytosis or receptor-ligand and increase the uptake of drugs by cells when they are inside the cell membrane. Different kinds, sizes, shapes, and electric charges have different effects on the entry of nanomaterials into cells. ③ Accelerate response to cellular environment and releases drugs. Different cell environments (ph, ROS, cytosolic reduction gradient, enzyme concentration) have different effects on nano-drug release.

In the current studies, the delivery of drugs to tumor cells by nano-carriers often involves three main processes: avoiding the elimination of drugs in the blood circulation when they are outside the cell, increasing the uptake of drugs by cells when they are inside the cell membrane, and accelerating response to cellular environment and releases drugs (The transfer process and influencing factors are shown in Figure 1). Particle size, surface charge, coating ligand, mechanical properties, and chemical properties, as well as route of administration, exhibit different biodistribution, compatibility, degradation, and cycling characteristics, thus have different effects on the anti-tumor process (Hardman, 2006; Schroeder et al., 2011). In the past decades, various types of nanomaterials have been reported for use in anti-tumor processes, and nanomedicine platforms have numerous different compositions and preparation methods. At present, the most widely used nano-drug delivery systems include liposomes, polymers, metals, carbon, biologicals, and so on. (Harisinghani et al., 2003; Voura et al., 2004; Larson et al., 2007; Heller et al., 2008; Lal et al., 2008; Horcajada et al., 2010; Ling et al., 2011; Wang et al., 2011; Qi et al., 2014; Maiolino et al., 2015; Xiao et al., 2015; Zanganeh et al., 2016; Shao et al., 2017; Cai et al., 2018; Patra et al., 2018; Akinc et al., 2019; Bayon-Cordero et al., 2019; Wu et al., 2019; Arshad et al., 2022; Subhan, 2022). (The advantages and uses of nanomaterials for medical diagnosis and treatment are shown in Table 2). Nanomaterials-based drug delivery systems (DDSs) are increasingly considered as a therapeutic option for MBC due to their ability to overcome the limitations of current therapies and provide good safety, controllability, and targeting. Nanoparticles (NPs) carriers currently show good results in cancer patients and various clinical trials.

**TABLE 2:** The advantages and uses of nanomaterials for medical diagnosis and treatment are shown in Table 2.

Materials	Types	Advantage	Function
Lipids	liposomes;	good biocompatibility;	drug delivery system;
	micelles;	low immunogenicity	reduced toxicity;
	liposome complex	good biodegradability;	enhanced efficacy
Polymers	polymer micelles	large payload	drug delivery system
	core-shell NPs	biodegradable	imaging analysis
	nanogels	excellent encapsulation performance	targeting circulating tumor cells
Metal Nanomaterials	metal NPs	good biocompatibility	drug delivery system
	metal nanorods	durability	imaging analysis
	iron-oxide NPs	convenient surface modification	photothermal therapy
	quantum dots	can be easily functionalized	
Carbon	carbon nanotubes	good biocompatibility and endocytosis	drug delivery system
	graphene	large surface area	monitoring drug concentration
Biologicals	protein NPs	low toxic side effects	drug delivery system
	nucleic acid NPs	high safety performance	
	Viruses		

## 4 Targeted drug delivery of novel nano-platform in BCLM

More and more novel nanoparticle-based targeted DDSs to improve the treatment of BCLM are being studied (Table 3) (Liu et al., 2018; Li et al., 2019a; Cheng et al., 2019; Xiong et al., 2019; Yu et al., 2019; Fan et al., 2020; Gong et al., 2020; Liu et al., 2020; Mu et al., 2020; Shi et al., 2021; Shi et al., 2021; Li et al., 2021; Zhang et al., 2021; Shi et al., 2023; Sabatelle et al., 2024). The process of DDSs to target the metastatic site can be divided into two steps, including one targeting the specific organ where the metastasis is located and the second targeting cancer cells or subcellular organs (Schroeder et al., 2011). At present, there are two strategies for targeted tumor delivery, one is passive targeting, and the other is active targeting. Passive targeting is the enhancement of the permeability and retention (EPR) effect of tumor vessels that leads to preferential aggregation of nanoparticles at tumor sites and increases tumor cell uptake. Active targeting refers to the ligand that binds to the receptor expressed on the nanomaterials, such as the design of albumin nanoparticles, so that the nanomaterials can bind to the receptor of cancer cells, and thus play a targeting role; on the other hand, it can respond to TME or stimulation and achieve the effect of controlled targeted release. Different approaches can maximize the drug’s ability to target and hit tumor cells accurately and reduce the drug’s interference on normal cells, to improve the effect of drugs and reduce the toxic side effects of the meaning of the body.

**TABLE 3:** Emerging nanoparticle-based targeted DDSs for direct drug delivery to secondary lung–tumor sites to improve the treatment of BCLM (Table 3).

Nanoparticles	Composition	Payload
nanocluster	Hyaluronic acid and BSA-coated gold nanocluster	PTX
biomimetic NP	RAW264.7 and 4T1 membrane, and PLGA	DOX
magnetic nanoparticle	Tetraethyl orthosilicate, tetrasulfide, acrylic acid, and cross-linker	DOX
nanoparticle	Polyglycerol Carbonate Nanoparticle	PTX
nanoparticle	hyaluronic acid-vitamin E succinate and poly (β-amino esters) (PBAEss) polymers	triptolide
liposomes	chondroitin sulfate-deoxycholic acid conjugates	DOX
nanoparticles	chondroitin sulfate-based with quercetin (chemosensitizer), chlorin e6 (photosensitizer)	PTX
nanoparticles	integrin α5(ITGA5) active targeting nanoparticles	ultrasmall platinum dot
biomimetic nanomaterial	erythrocyte membrane (M)-camouflaged graphene oxide quantum dots	DOX
nanoparticles	poly-N-(2-hydroxypropyl) methacrylamide (pHPMA)-coated wheat germ agglutinin-modified lipid-polymer hybrid nanoparticles	Silibinin and cryptotanshinone
nanoparticles	scalable drug-combination nanoparticle platform	gemcitabine and paclitaxel
biomimetic nanoparticles	photosensitizer pheophorbide A	paclitaxel dimer prodrug
cell-like nano-prodrug	DNA tetrahedron dendrimer with a liposome and macrophage membrane	DOX
nanoparticles	RAW 264.7 cells with laurate-functionalized Pt (IV) prodrug, human serum albumin, and lecithin	cisplatin
nanoparticles	glycol chitosan (GCS) formed nanogels	Suramin and DOX

### 4.1 Passive targeting based on nanotechnology

After NPs are absorbed in different ways, they circulate in the blood and will be distributed to different organs and tissues. In solid tumor tissues, there are abundant blood vessels, wide gaps between blood vessel walls, poor structural integrity, and absence of lymphatic reflux, which make macromolecular materials have higher permeability and retention, this phenomenon is called the enhanced EPR effect. In large primary tumors and their secondary metastases, the EPR effect enables nanomaterials to accumulate and be retained by the tumor. For giant primary tumors and their secondary metastases, nanoparticles circulating in the blood can extravasate through leaky blood vessels at the tumor site, and the EPR effect enables nanomaterials to accumulate and be retained by the tumor. Nanoparticles circulating in the blood can extravasate through leaky blood vessels at the tumor site and accumulate in large, well-vascularized tumors. Due to poor lymphatic drainage, the particles remain at the tumor site. The intravenous injection was the common way for pulmonary tumor targeting delivery via the EPR effects. However, small metastases (>100 mm^3^ in volume) were poorly vascularized, and nanoparticles could not enter well through EPR effect. The EPR effect is limited to a certain extent, so it is still a great challenge to transfer the DDSs to the site by passive targeting (Schroeder et al., 2011). At the same time, some researchers now suggest that passive extravasation accounts for only a small fraction of nanoparticle tumor accumulation and that the EPR effect can be observed in mice, but may not have the same effect in humans, and the heterogeneity of the tumor may disturb the EPR effect (Sindhwani et al., 2020). However, the theory of the EPR effect is often used in the design of drug delivery systems as a part of the delivery process. Even though some of the properties of NPs, a prime example being liposomes, result in their short blood circulation time, researchers have altered the physicochemical properties of nanosystems by adding polyethylene glycolas as a surface coating, the combination of the EPR effect may be effective in reducing the effect of defects that are quickly eliminated (Bourquin et al., 2018).

### 4.2 Response controlled drug-releasing strategies of targeting

After the nano-drug reaches the tumor site, the effective control of drug release becomes a key issue. The drug release rate can significantly affect the therapeutic effect. Some studies show that rapid release of intracellular drugs can help overcome chemoresistance and kill tumor cells (Gao et al., 2011). The stimulus-response strategy provides a useful approach for site-specific controlled drug release. Stimulation-responsive nano-carriers produce physical or chemical changes, such as molecular chain structure, surface structure, swelling, solubility, and dissociation, when stimulated by external signals. The TME is characterized by its abnormal conditions, such as acidic pH, enhanced reactive oxygen species (ROS), cytosolic glutathione (GSH), cytosolic reductive gradient, hypoxia, and over-expressed proteases, which often act as endogenous triggers to stimulate the release of nanomedicine (Mbeunkui and Johann, 2008). In addition, exogenous stimuli can also be used for controlled drug release, such as temperature, light, ultrasound, external alternating magnetic fields, and electric fields. Based on the different nature of tumor cells and TME from normal cells or tumor cell targets, a specific carrier system is designed to achieve active drug enrichment to tumors and TME. Nanotechnology has great potential application value in the treatment of tumor metastasis. To minimize systemic toxicity, many attempts have been made to utilize stimulatory-reactive therapeutics that promise to selectively activate pathological tissues by exploiting the TME.

#### 4.2.1 Ph responsive

PH-reactive DDSs are often designed to make carriers stable in different environments, targeted drug release occurs when drug-loading systems enter the tumor extracellular microenvironment or more acidic intracellular lysosomes, while they remain stable in the blood (He et al., 2015; Wang et al., 2018). This drug will be released in a specific environment, on the one hand, to enhance drug targeting, on the other hand, to reduce the toxic damage to normal tissue. The engineered NPs can achieve the precise release of anticancer drugs through the pH response pathway for precision therapy. A pH-responsive host-guest nanosystem of succinobucol with pH-stimuli controlled drug release behavior, which was composed of a host polymer with a guest polymer and active agent of butanediol succinate, has showed specific and rapid drug release to intracellular acidic pH stimulation and has been shown to significantly improve the treatment of lung metastases from breast cancer (Dan et al., 2016). Ph-sensitive nanoparticles are used for the co-delivery of docetaxel (DTX) and dihydroartemisinin (DHA), rather than the traditional drug delivery model, reduce side effects by releasing tumor-specific drugs in response to an acidic environment. The results showed that the nanoparticles inhibited tumor growth and prevented lung metastasis in an *in situ* metastatic tumor mouse model derived from 4T1 cells (Tao et al., 2020). Succinol-loaded pH-responsive helminth-like micelles with continuous targeting capacity can not only be delivered specifically to sites of metastasis in the lung but can also undergo pH-stimulated responsive drug release within cancer cells to significantly inhibit the migration and invasion of metastatic 4T1 breast cancer cells (He et al., 2015). The sequential targeting of the nano-drug delivery system based on the principle of drug action pathway and the characteristics of pH has been proven to be a promising strategy for the treatment of BCLM. The pH response is also utilized in the process of charge reversal nano-drug delivery system. The nano-carriers remain indolent in the blood circulation process, and reach the TME through the EPR effect in solid tumors, and then are activated by the low pH of the TME, positively charged nanoparticles interact more strongly with cell membranes (negatively charged surfaces) to increase cellular uptake and thus enhance drug efficacy (Oupicky et al., 2002; Arvizo et al., 2010). A growing number of nano-delivery platforms are designed to take full advantage of the differential pH environment of the tumor, allowing pH-sensitive responses to be involved in the drug release process for more refined targeting.

#### 4.2.2 Redox type responsive

Redox DDSs typically take advantage of the large differences in ROS levels in the TME and the abundance of GSH, enabling the generation of selective activation effects. The concentration of GSH in the cytosol was 1–10 mM, about 100–1000 times that in plasma. Typical ROS levels can be as high as 100 μm in cancer cells and as low as 20 nm in normal tissues (Hu and Liu, 2020). *In vitro* and *in vivo* experiments, the site-specific drug release effect was achieved by using different GSH contents in tumor ecology. Using the Kendall effect and electrostatic attraction to form an ultra-high drug-loading nano-delivery platform, which is modified by coating the tumor cell membrane to precisely enter the tumor site, where they respond to overexpressed GSH. Then O_2_ production and GSH consumption during the internal response reinforce external photodynamic therapy, and the heat generated through photothermal therapy during the external response promotes internal chemodynamic therapy (Xu et al., 2021). Shi et al., 2023 have successfully constructed a pH/redox dual-responsive magnetic nanoparticle to deliver doxorubicin (DOX), which has been efficiently internalized into tumor cells and subsequently triggers drug release in response to changes in GSH concentration and pH, demonstrating anti-metastatic efficacy *in vivo* in a model of lung metastatic breast cancer. Despite the great achievements in the design of GSH-responsive nanocarriers, we should note that despite the significant differences in GSH concentrations in plasma and cytosol, GSH levels in healthy and cancer cells were comparable (Hu and Liu, 2020). Nano-platform FA-BSA@DA has been developed to inhibit breast cancer metastasis by targeting heparanase, with folic acid modified to enhance the targeting of lung metastases. After activation of versatile aspirin prodrug DA by H_2_O_2_ in the tumor, aspirin is released, the activity of heparanase is inhibited, and the metastasis of cancer to the lung is further inhibited (Zhang et al., 2020). Experimental studies have shown that many of these nanocarriers are captured in the liver and spleen, and the accumulation and uptake of these nanocarriers in pathological tissues remains limited, leading to unsatisfactory treatment outcomes. In general, the practical application of redox-responsive nanocarriers still needs more exploration. Amplification of endogenous redox signals and enhancement of the selectivity of targeting tumor tissues have shown more research value and become crucial.

#### 4.2.3 Enzyme responsive

After the nanocarriers reach the TME, they are recognized and sheared by enzymes specifically to expose the encapsulated drugs, and become nanocarriers in response to enzyme stimulation in the TME. The expression levels of specific enzymes, such as protease, glycosidase, and phospholipase, are increased in the tumor environment. Therefore, the difference between the tumor tissue and the normal tissue environment can be utilized, to design and construct the nano-drug delivery system for enzyme environmental response. In recent years, enzyme-responsive nanoparticle systems have become a research hotspot and more and more scholars focus on lysosome-related studies. The proliferation and metastasis of tumor cells are closely related to lysosomal enzymes. After nanoparticles enter the lysosome through endocytosis, they undergo depolymerization or degradation, which accelerates the process of drug release, thus significantly increasing drug accumulation in cells (Ichihara et al., 2007; Bertout et al., 2009; Lee et al., 2011; Singh et al., 2015). Legumain-activated nanoparticles, which conclude the anti-cancer drug of mertansine is conjugated to poly with a legumain-sensitive peptide linker, are loaded into Ly6c + inflammatory monocytes to improve targeting ability to lung metastases of breast cancer. The differentiation of monocytes into macrophages in the metastatic niche and response to a highly expressed legumain protease environment, anticancer drugs can be isolated to damage macrophages and released to the metastatic site (He et al., 2017). The enzyme environment of metastatic tumors is very complex and diverse. The exploration of potential enzyme activity may bring new therapeutic hope for BCLM.

#### 4.2.4 Exogenous stimuli responsive

Exogenous stimuli can also be used to control drug release, including light, ultrasound, magnetic fields, temperature, and other stimuli, mainly using special physical and chemical principles. The nano-system can be used for controlled drug release in response to specific external physical stimuli applied to the tumor, and can also assist in imaging or therapeutic functions. It has been developed for light-mediated drug delivery and aptamer-targeted cancer therapy. Using graphene oxide composite microcapsules, Kurapati et al. (Kurapati and Raichur, 2013) demonstrated a near-infrared (NIR)-light-controlled-release drug pathway. After laser irradiation, the graphene oxide-polymer complex capsules were opened in a“point-by-point” manner under remote stimulation due to local heating, and the release of DOX, an anticancer drug, was successfully applied. Nano-reagents can generate heat under NIR light irradiation, which is used to initiate photothermal therapy (PTT) to directly kill tumor cells (Yang et al., 2015). Therefore, the nano-platform can not only effectively control the time and location of the targeted delivery and drug release under exogenous stimulation, but also it is often used in the diagnosis and treatment of tumors together with imaging function and photothermal therapy.

### 4.3 Functionalization modified type of active targeting

The basic concept of a functional nanosystem is to selectively target drugs to cancer cells by modifying the surface of nanoparticles to maximize drug effects and reduce undesirable damage to normal cells (Sun et al., 2014). At present, many studies have designed nanomaterials with ligands that can bind to receptors expressed on tumor cells for targeted targeting. Antibody-based targeting ligands have been used on various nano-delivery systems.

Li et al. (Li et al., 2019b; Li et al., 2021) have taken advantage of the properties of ITGA5, which is highly expressed in TNBC cells and their lung metastases, to active targeted drug delivery to TNBC cells via ITGA5 ligands, such as a commercialized ligand-RGD motif (ARG-GLY-ASP). The results demonstrated that RGD-modified lipid-polymer hybrid nanoparticles accumulated more significantly and stayed longer in breast TNBC tumors and lung metastases *in situ* in nude mice. They also subsequently reported the integrin ITGA5 active targeting nanoparticles, which have loaded a novel ultrasmall Pt (II) dot from miriplatin. Severe DNA damage induced by Pt (II) dot NPs activated the Chk1/2-CDC25A-cycline A/E pathway to induce cell cycle arrest. It can also target CTCs *in vivo* to inhibit lung metastasis of TNBC. Tumor-specific functionalization-modified NPs partially facilitate drug entry into the tumor, resulting in increased local drug concentrations at the primary and metastatic sites, while off-target effects are reduced. However, current breast cancer-specific targeting agents may not be able to provide accurate and reliable true full-specificity targeting, as some biomarkers are also expressed in normal healthy tissues. Reliance on only one biomarker can lead to erroneous targeting, such as transferrin receptor expression in both cancer cells and some fast-growing cells. It needs more perfect research work to explore more accurate receptor targeting based on nano-platform.

## 5 Application of nanotechnology in the diagnosis and treatment of BCLM

**FIGURE 2:**
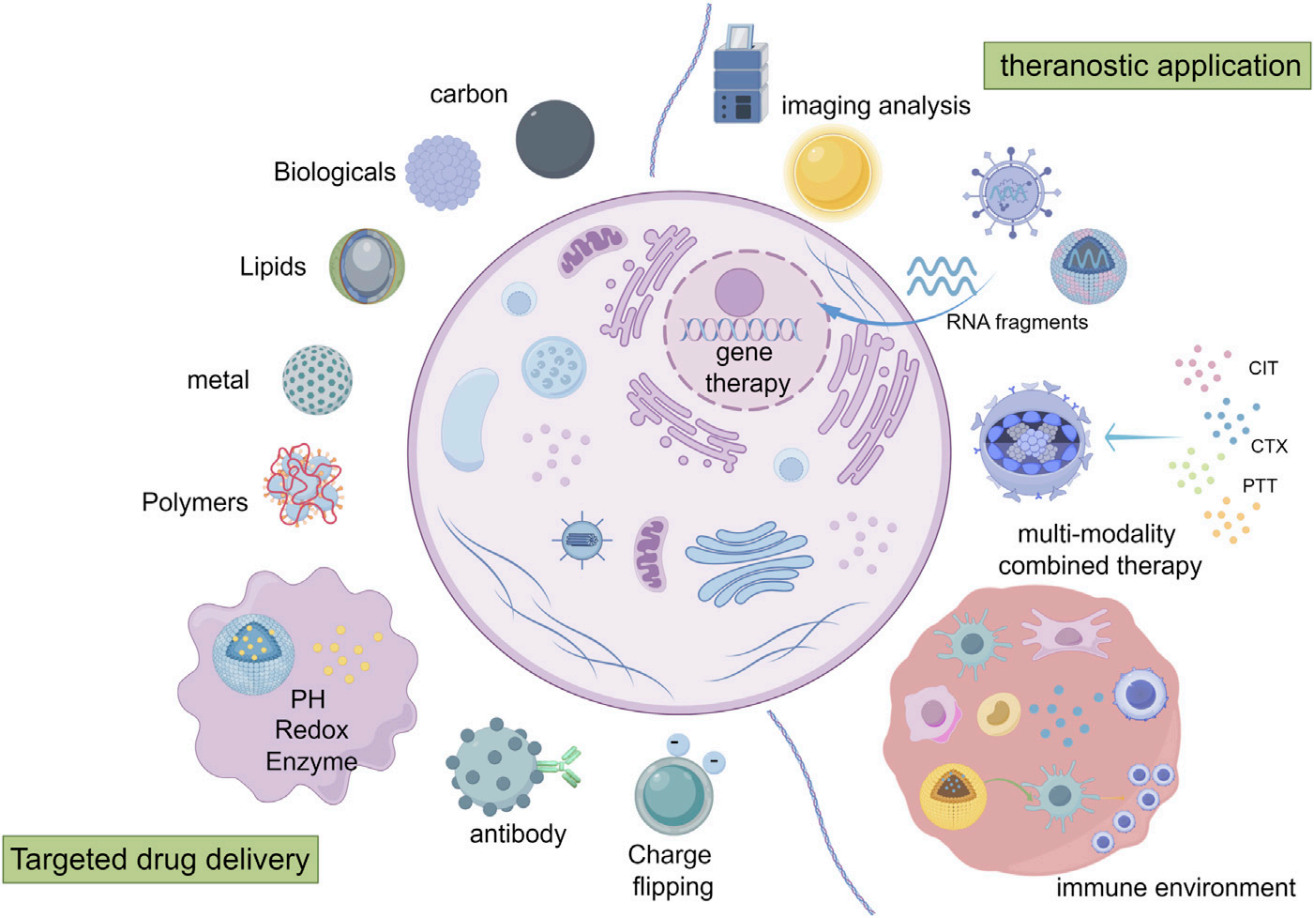
Mechanisms of targeted drug delivery and application of nanotechnology in diagnosis and treatment of BCLM (By Figdraw). ① drug delivery: the delivery function can be achieved by passive targeting, functional modification, active targeting and responsive targeting. ② imaging analysis. ③ immunotherapy/genetherapy/multiple treatment methods combined therapy: the nano-system carries on the precision treatment to the tumor and the tumor microenvironment.

In recent years, the mode of early diagnosis for advanced breast cancer has been opened up, the drug treatment scheme has been updated, and the surgical scheme has been explored, but the treatment dilemma of BCLM has not been solved. New therapeutic methods, such as immunotherapy and gene therapy, have entered the stage of rapid development and brought new hope for the treatment of tumors. Among them, is the use of nanotechnology for a variety of new ways to provide assistance and play an irreplaceable role. (Nanotechnology applications in the diagnosis and treatment of BCLM in Figure 2).

### 5.1 Application of imaging analysis in breast cancer lung metastasis

Early and accurate diagnosis of BCLM creates favorable conditions for subsequent treatment, but high-standard imaging diagnostic methods are limited at present. The use of nano-contrast agents may overcome the limitations inherent in current imaging and make targeted imaging possible. For patients with metastatic cancer, the development of nano-drug-loading technologies has the potential to reduce the toxicity of imaging agents while improving specificity, signal, and strength, and to enable visualization of metastases during surgery. Nano-drug loading is helpful for clinical diagnosis and treatment simultaneously, which is the development trend in the future (Wu et al., 2010; Pan et al., 2014; Khandelia et al., 2015; Zheng et al., 2018).

Metal oxide Magnetic nanoparticles are the most widely studied and used contrast agent in tumor imaging. These particles have excellent biocompatibility, reduced cytotoxicity to chemotherapy drugs, and remarkable ability to act as magnetic resonance imaging (MRI) contrast agents, it has led to advances in molecular and cellular imaging, cancer therapy, and integrated nanoscale devices for cancer detection and screening. For MRI, superparamagnetic nanoparticles made of iron oxide can produce higher contrast at lower concentrations than gadolinium, a common MRI contrast agent. Iron oxide nanoparticles have developed into an effective tool for diffusion imaging of metastatic cancer. The FDA (US Food and Drug Administration)-approved iron supplement FERUMOXYTOL and other iron oxide nanoparticles have been used as magnetic resonance imaging and drug carriers, ferumoxytol has an intrinsic therapeutic effect on the growth and liver and lung metastasis of early breast cancer. Nanoscale organometallic scaffolds act as drug delivery carriers, Mn_2_^+^ as encapsulating diagnostic developer, and DOX as therapeutic compounds to achieve accurate visualization of nanocarrier accumulation, MRI is used for accurate tumor localization, diagnosis, and imaging-guided treatment. In addition, functional MOFs-based nanocarriers have shown effective ablation of primary breast cancer, as well as significant inhibition of lung metastasis and high survival (Harisinghani et al., 2003; Zanganeh et al., 2016; Shakil et al., 2019; Meng et al., 2021). More and more nanotechnology has been developed and matured in the application of tumor imaging, showing its unique advantages.

### 5.2 Nanoparticles for immunotherapy against breast cancer lung metastasis

With the development of tumors and the role of the immune system, immunotherapy has become a promising therapeutic strategy. The development of the tumor enters the “escape” stage, and the balance between the tumor and the immune system is broken, the TME recruits myeloid suppressor cells, cancer-associated fibroblasts, tumor-associated macrophage cells and regulatory T cells, and produces immunosuppressive cytokines and soluble factors, thus the immunosuppressive tumor microenvironment is established. Immune evasion in cancer may arise from multilayer immunosuppression in the TME, and the expression of immune checkpoint molecules on the T lymphocyte may also inhibit the effect of anti-tumor immunotherapy (Liu et al., 2019). TNBC usually has higher levels of apoptotic process ligand (PD-L1) expression than other subtypes of breast cancer. Keynote 522, Keynote 355, and others have shown that immune checkpoint inhibition in triple-negative breast cancer confers a survival benefit, and triple-negative breast cancer is currently the only type of breast cancer that has achieved clinical survival improvement in immunotherapy studies (Cortes et al., 2020; Schmid et al., 2020; Banerjee and Rajeswari, 2023).

Tumor immunotherapy brings new hope for refractory breast cancer, and the application of nanotechnology can overcome the barrier of immunotherapy to some extent. The activation of dendritic cells by gold nanocomposites containing Ganoderma lucidum polysaccharides (GLP-AU) constructed by Zhang et al., 2019, the results indicated that GLP-Au combined with adriamycin could inhibit lung metastasis. Duan et al., 2016 have reported Zn-pyrophosphate (ZnP) nanoparticles loaded with the photosensitizer pyrolipid (ZnP@pyro) to kill primary 4T1 breast tumor and inhibit lung metastasis, which improved checkpoint blockade immunotherapy works by activating the innate and adaptive immune system parts of the TME. Interleukin-12(Il-12) acts both directly and indirectly by recruiting and activating other immune effectors as well as secreting cytokines, and Lai et al. used LNP-encapsulated IL-12 mRNA to have good antitumor efficacy in a liver tumor model. Administration of Il-12 has previously been shown to have therapeutic benefits in preclinical models of breast tumors, so we can further explore the potential therapeutic implications of nanotechnology-based approaches (Lai et al., 2018). It is important to understand the mechanism of immunity and treatment for advancing nanotechnology in the treatment of advanced metastatic breast cancer.

As an important part of immunotherapy strategy, tumor vaccine, which aims to stimulate a tumor-specific immune responses, has shown an unprecedented prospect with the application of nanomaterials in this field. In the immunocompetent mouse model, the nano-vaccine significantly inhibited the growth and development of 4T1 breast tumors (Zhou et al., 2020). However, there are still some problems, such as instability during delivery, insufficient drug entrapment efficiency, and insufficient drug release, etc., so it is necessary to optimize the efficacy of the vaccine and select the appropriate treatment strategy.

### 5.3 Nanoparticles for gene therapy against breast cancer lung metastasis

In most cancers, the immune microenvironment is a balance between immune cells that mediate and prevent tissue destruction. Gene therapy aims to treat the disease by regulating apoptosis and cell function at the genetic level and often introduces foreign genes into target cells to treat diseases caused by gene defects or abnormal gene expression. However, siRNA, miRNA, and other therapeutic agents are easy to be degraded *in vivo*. How to deliver therapeutic genes to tumor cells safely and effectively is a problem to be solved before the clinical application of tumor gene therapy.

SiRNA can indirectly regulate gene expression by fine-tuning the blocking or downregulation of many different kinds of genes in the body. ONPATTRO, an LNP formulation of siRNA, was approved by the FDA in 2018 (Akinc et al., 2019). Sakurai et al. (2018) have delivered siRNA via RGD peptide-modified lipid nanoparticle (RGD-lnp), leading to a significant silencing in the metastasized vasculature. This experiment significantly prolonged the overall survival of the metastatic model mice for the treatment of metastatic tumors. MiRNA can regulate multiple genes simultaneously, thus regulating multiple processes, which are considered promising therapeutic targets for cancer metastasis (Kong et al., 2018; Gan and Gunsalus, 2019). Liu et al. (2016) delivered Map3k1-amiRNA into 4T1 breast cancer cells using a miRNA-expressing lentiviral system and showed that MAP3K1 amiRNA attenuated tumor growth and lung metastasis of breast cancer cells *in vivo* nude mice model. Jiang et al. (2023) have constructed DNAzyme nanocapsules, which encapsulate poly -LRB-ethylenimiPEI (PEI)-DNAzyme iMN2 MnZN2/Zn2 +-coordinated inositol hexaphosphaIP6(IP6) capsule modified with cRGD targeting peptide, exhibits effective therapeutic efficacy against both primary and metastatic breast tumors inMCF-7CF-7 breast tumor-bearing mouse model. In this study, apoptosis was induced by downregulation of EGR-1 and BCL-2 protein expression. TP53 is the most commonly mutated or deleted gene in TNBC, and Polr2a in the TP53-adjacent region is an incidental frailty target for TNBC tumors. The pH-responsive nanoparticle that targeted Polr2a has therapeutic potential in TNBC with common TP53 genomic alteration (Xu et al., 2019).

At present, there are two kinds of effective gene therapy: immune cell-based therapy (CAR-T) and oncolytic virus-based gene therapy (OVS). Nanoparticles, as delivery vectors of gene therapy components, have been widely studied in anti-tumor therapy, and may be used in some potential therapies for breast cancer with lung metastasis in the future, however, there is still a lack of clear clinical research conclusions. Nanoscale-based, multi-pronged approaches to cancer treatment combine autologous immune enhancement therapy with traditional therapies and other approaches such as hyperthermia and proton therapy, provide interesting feedback.

### 5.4 Nanoparticles for multi-modality combined therapy against breast cancer lung metastasis

Combination therapy is becoming more and more common in anti-tumor treatment. Clinicians use combination therapy, such as surgery plus chemotherapy, chemotherapy plus radiotherapy, and so on, to achieve better therapeutic effects. Researchers are also focusing more research on combination therapies, which yield better results than single drugs or treatments. Combination therapy is often considered a promising strategy.

Chen et al. (2014) reported a novel therapeutic “Abraxane-like” nanosystem composed of human serum albumin, paclitaxel, and indocyanine green, it has been shown to have a significant therapeutic benefit in mice with metastatic tumors following a highly efficient combination of photothermal and chemotherapy guided by near-infrared imaging. The combination of photothermic and chemotherapeutic therapies offered by nanoscale therapeutic agents has attracted great attention in recent years. Liu et al. (2018) have used tumor-specific CD44-targeted, hyaluronidase-degradable hyaluronic acid (HA) and small-size, kidney-scavenger, red-emitting, bovine serum albumin-protected gold nanoclusters (AuNC@CBSA) to successfully build a size-reducing nano-platform (AUNC@CBSA@HA), and load paclitaxeland indocyanine green for chemophotothermal therapy. The constructed AUNC@CBSA-PTX-ICG@HA-NO3 nano-platform showed inhibition of 95.3% *in situ* tumor growth and 88.4% lung metastasis growth. Zhang et al. (2019) have designed a nanoparticle that integrates three FDA-approved therapeutic agents, Indocyanine Green (for PTT), Adriamycin (for chemotherapy), and CpG (for immunotherapy), into layered hydroxide (LDH). In a 4T1 breast cancer model, this multifunctional IDCB-LDH nanomedicine effectively eliminated primary tumor tissue, prevented lung metastasis, and inhibited tumor growth with distant revaccination. It provides specific preparation for the combination of PTT, chemotherapy, and immunotherapy. Wang et al. (2023) have designed a biodegradable lipid-camouflaged bismuth-based nanoflowers, and configured it with dry powder technology to better achieve lung accumulation while the nanoflowers deposited in the tumor area and burst to release, lower distribution of the whole body. The results show that it has good photothermal conversion efficiency, enhanced radiation therapy and CT imaging ability to effectively achieve the synergistic effect of PTT and radiotherapy at the same time in lung metastasis of breast cancer, which inhibits tumor cell invasion and metastasis with inhibition of snail and N-cadherin expression.

Because of the multi-factors and multi-steps involved in the process of anti-tumor treatment, the single treatment may not be able to achieve satisfactory results. A growing number of treatment regimens favor a combination of two or more treatments. Nanosystems are good options, not only for specific drug targeting but also for simultaneous delivery of drugs and delivery of combination therapy, providing a good foundation for the development of combination therapy approaches in BCLM.

### 5.5 Clinical applications of nanotechnology in breast cancer lung metastasis

More and more clinical trials focus on the application of nanotechnology in BC and MBC and continuously obtain positive results and feedback. Doxil, Abraxane, and other nano-drugs with different physical properties and biological effects have been used in clinical trials (Park et al., 2022). Early clinical trials have shown that nanoparticle albumin-bound (nab-) paclitaxel is more effective than conventional solvent-based (sb-) paclitaxel in the treatment of MBC. Albumin-bound (nab-)paclitaxel ABI-007, which is approved to have anti-tumor ability in patients with metastatic breast cancer, has been recently approved by the FDA for pretreated MBC patients (Miele et al., 2009; Lee et al., 2020). A phase II, multicenter, single-arm study studied the acceptable safety of low doses of 180 mg/m^2^ nab-paclitaxel in patients with metastatic or recurrent breast cancer and concluded that this may be an effective therapeutic way (Yamamoto et al., 2017). Although conventional anthracyclines have proven effective for metastatic tumors, they are often accompanied by adverse cardiotoxicity. A phase I, single-arm study in MBC patients who were naive to previous chemotherapy studied the effect of the toxicity of nab-paclitaxel/nonpegylated liposome-encapsulated doxorubicin combination, and confirmed that this is an effective regimen with mild toxicity, which deserves confirmation by larger tests (Fabi et al., 2020). Atezolizumab in combination with nab-paclitaxel has been approved, which has therapeutic significance for patients with metastatic TNBC in the PD-L1 positive subgroup (Schmid et al., 2018). Nanoparticle-based DDSs are now continuously exploring and demonstrating strong potential in MBC chemotherapy protocols.

At present, the application of nanotechnology to lung metastasis of breast cancer is mostly limited to preclinical research but has shown great advantages and potential. There is still a long way to go before nanotechnology can be used in clinical practice in advanced breast cancer. In this process, researchers need to consider much more than mechanism and biosafety. While breakthroughs in improving patient outcomes remain to be achieved, these clinical trials show researchers and clinicians that nanotechnology may be a potential therapeutic modality for BCLM.

## 6 Conclusion and outlook

Lung metastasis is the main cause of death in cancer patients, but there is still a lack of effective treatment for lung metastasis. Traditional treatment methods often lead to the failure of treatment and patients’ intolerance because of drug resistance, systemic tissue toxicity, and lack of effective targeting. New options are therefore urgently needed to overcome the challenges of BCLM-related therapies, particularly in terms of tumor targeting.

In this review, we focus on the application of drug-loaded therapeutic nanoparticles in the treatment of BCLM, specifically targeting the TME as a complex but promising research area. It is not difficult to find that nanoplatform-based therapies for BCLM may have higher efficacy and lower toxicity. At the same time, nanotechnology has shown unique advantages in the immunotherapy and gene therapy of BCLM. More and more researchers will focus on the search for BCLM targets with higher specificity, the use of multi-target synergy to improve the targeting ability, the functionality, and applicability of nanomaterials. Although many preclinical studies have provided new therapeutic ideas and obtained promising results, the clinical transformation of nanoparticles for the treatment of BCLM has been limited so far.

At present, it is difficult for nano-drugs to develop further into clinical practice, which mainly involves the instability and cost of the manufacturing process. At the same time, it is a long and complicated process for researchers to select suitable drug-carrying materials, not only considering the physical chemistry properties of the materials themselves but also there is a need to give serious consideration to biosafety issues that are closely linked to the human body.

In the research of cancer therapy based on nano-systems, the ideal state is that the nano-DDSs are only effective for tumor cells and TME, but this is a difficult process because the mechanisms of tumor cell mutation, tumor metastasis and tumor heterogeneity are not clear yet. Understanding of the pathogenesis of BCLM will also be enhanced in the development of applications targeting nanomedicine, the process that will also accelerate the development of effective BCBM therapy nanomedicine. Currently, the major challenge for nano-drug development is how to achieve complete elimination of the primary tumor and the prevention of tumor metastasis with limited nanomedicine delivery. To date, most cancer nanotechnology therapies have focused on the treatment of primary tumors, but it is important to harness the potential of nanotechnology to fight the spread of cancer at every stage of the metastatic process. We need to devote more effort to further study the valuable practice of nano-platform in BCLM.
